# The Combination of Enzymes and Conidia of Entomopathogenic Fungi against *Aphis gossypii* Nymphs and *Spodoptera frugiperda* Larvae

**DOI:** 10.3390/jof10040292

**Published:** 2024-04-17

**Authors:** Juliana M. Ferreira, Éverton K. K. Fernandes, Jae Su Kim, Filippe Elias F. Soares

**Affiliations:** 1 Institute of Tropical Pathology and Public Health, Universidade Federal de Goiás, Goiânia 74605-050, Brazil; julianamarqueslv@gmail.com (J.M.F.); evertonkort@ufg.br (É.K.K.F.); 2 Department of Agricultural Biology, Jeonbuk National University, Jeonju City 54896, Republic of Korea; jskim10@jbnu.ac.kr; 3 Department of Chemistry, Universidade Federal de Lavras, Lavras 37200-900, Brazil

**Keywords:** biological control, entomopathogenic fungi, enzymes, pest management

## Abstract

The slow action of fungi is one of the biggest challenges in using entomopathogenic fungi. A promising alternative to reduce the time of action is to combine conidia with extracellular enzymes. This study aimed to characterize the production of Pr1 subtilisin protease and lipases by *Beauveria bassiana* and *Metarhizium anisopliae* in different culture media and to evaluate the efficiency of the enzymatic treatment against *Aphis gossypii* and *Spodoptera frugiperda*. The isolates were cultivated in five different liquid cultures, and, after 7 days, the culture was filtered and centrifuged, and the activity of the Pr1 and lipases was measured. The fungi cultured in a Luria–Bertani broth medium had the highest activity of proteases and lipases. The mortality of *A. gossypii* nymphs treated with conidia 7 days after the treatment was 39% (JEF-410), 76.5% (JEF-492), 74.8% (ERL-836), and 70.9% (JEF-214). The *B. bassiana* JEF-410 supernatant combined with conidia increased the fungal virulence at day 5 and day 6 after treatment. When *S. frugiperda* larvae were treated with *B. bassiana* JEF-492 conidia combined with its supernatant, the time of infection was shorter compared to the larvae treated with conidia only. Once the supernatant was incubated at 37 °C, the relative activity decreased from 100% to 80% after 2 h and to 45% after 24 h. The results suggest that the supernatant of entomopathogenic fungi may be formulated and used as a biopesticide in an efficient strategy for the biological control of pests.

## 1. Introduction

Studies have investigated the use of entomopathogenic fungi as biological agents to establish rational and effective strategies for arthropod control. Fungi such as *Metarhizium anisopliae* (Metschn.) Sorokin, 1883 and *Beauveria bassiana* (Bals.-Criv.) Vuill., 1912 complexes have proven highly effective against various arthropod species [1]. Entomopathogenic fungi, such as *B. bassiana*, are effective biocontrol agents against many agricultural pests [2], as well as baculoviruses and bacteria including *Bacillus thuringiensis* [3]. In Brazil, the most extensive microbiological control program utilizes *M. anisopliae* s.str. against spittlebugs in sugarcane crops [4]. Also, *B. bassiana* controls whiteflies, coffee berry borer, and other agricultural pests [5].

The primary mechanism of fungal infection against insects is through the cuticle, unlike other pathogens that enter the insect hosts by ingesting contaminated food [6]. The penetration process through integument occurs via the degradation of the cuticle due to the action of several enzymes—such as proteases (EC 3.4) and lipases (EC 3.1.1.3)—combined with the mechanical pressure of the hyphae [7]. The fungal penetration continues through all the integument layers until the hyphae reach the hemocoel [8].

The insect’s cuticle is the first barrier against microbial invasion. The external layer, the epicuticle, is hydrophobic and mainly composed of lipids, fatty acids, esters, and long-chain alkenes [1]. Therefore, lipases are produced during the initial infection process and are responsible for the hydrolysis of the ester bonds of fats, waxes, and lipoproteins from the epicuticle [9].

Subsequently, the fungus has to penetrate the host through the procuticle—a proteinaceous layer. The insect’s cuticle is mainly constituted of proteins (up to 70%), and, for this reason, proteases are one of the most vital enzymes related to the infection process [1]. Among the subtilisins, trypsins, metalloproteases, and exopeptidases, Pr1(subtilisin) is mainly responsible for the degree of pathogenicity of the isolate [1,9,10]. It can be found in 11 isoforms (Pr1A-K), each with a distinct participation in the infection process [11].

The slow action is one of the most significant challenges in using entomopathogenic fungi. Fungi, as biopesticides, usually take more days to eliminate the insects than chemical pesticides, which may represent economic losses to farmers [7]. One alternative to reduce the time of infection is to combine entomopathogenic fungi with chemical pesticides [12,13]. However, combining fungi with biochemical pesticides may be a more sustainable way to use them.

A new approach to exploring entomopathogenic fungi as biopesticides involves using their extracellular enzymes to accelerate the time of infection. Studies have tested fungal enzymes as biopesticides with promising results, as reviewed by [7]. As the enzymes act in the degradation of the arthropod cuticle, the time of infection can potentially be decreased if they are applied along with fungal conidia [14]. Bioproducts are promising alternatives to the exclusive use of synthetic pesticides because the overuse of these chemicals, including organophosphates, carbamates, and pyrethroids, may select resistant insect populations [15].

*Aphis gossypii* (Glover) (Hemiptera: Aphididae), also known as the cotton aphid, is a sucking pest that causes physical damage to the plant not only by feeding but also by transmitting viruses and producing honeydew excretion. *A. gossypii* is a polyphagous pest found worldwide that feeds on several plants, such as vegetables, fruits, and flowers [16]. The fall armyworm (FAW) or *Spodoptera frugiperda* (J.E. Smith, 1797) (Lepidoptera: Noctuidae) is a highly polyphagous insect that feeds on leaves during the larval stage [3]. It prefers graminaceous plants, such as maize, sorghum, barley, and wheat. It can also eat other plants, including cotton and soybean [17]. *S. frugiperda* is a destructive pest in the Americas and recently arrived in Africa (2016–2017), Asia (2018–2019), and Oceania (2020) [18]. It is estimated that the FAW causes losses of about USD 300 million in the USA and USD 13 billion across sub-Saharan Africa per year [17].

This research aimed to characterize the production of protease Pr1 and lipases from different isolates of entomopathogenic fungi in various culture media and to evaluate the effectiveness of enzymatic treatments associated with conidia against different insect species of agricultural relevance.

## 2. Materials and Methods

### 2.1. Fungal Isolates

Fungal isolates were provided by the Jeonbuk National University Entomopathogenic Fungal Platform (JEF-LIBRARY). Four isolates were selected: three *B. bassiana* (JEF-410, JEF-492, and ERL-836) and one *M. anisopliae* (JEF-214). The isolates were collected from soil using the *Tenebrio molitor* larva-based baiting method. The collection sites were as follows: JEF-410 (Jeongseon, Republic of Korea); JEF-492 (Jeongseon, Republic of Korea); ERL-836 [CA, USA (original isolation code: CA13)]; and JEF-214 (Pyeongchang, Republic of Korea).

The fungal isolates were cultivated on one-quarter of Sabouraud dextrose agar (SDA, BD Difco, Sparks, MD, USA) at 27 ± 1 °C in the dark for 14 days. Five culture media were prepared: a Luria–Bertani broth (LB), a potato dextrose broth (PDB), a Sabouraud dextrose broth (SDB), a yeast extract–peptone–glucose broth (YPG), and a soluble starch–yeast extract–peptone broth (SSYP). All culture media had the initial pH adjusted to 6.0 [19].

Conidial suspensions of each fungal isolate at 1 × 10^7^ conidia mL^−1^ (1%, *v*/*v*) were inoculated in 100 mL liquid cultures. The cultures were incubated on an orbital shaker at 27 ± 1 °C and 170 rpm for 7 days. After this period, the liquid cultures were filtered through filter paper (No. 2 Ø110, ADVANTEC, Tokyo, Japan). The filtrate was then centrifuged at 10,000 rpm for 6 min at 4 °C, and the supernatants were stored at 4 ± 1 °C. The PDB medium was used as the control medium [19].

### 2.2. Enzyme Assay

The activity of the subtilisin-like Pr1 protease in the culture supernatants was measured by a method adapted from [19,20]. The substrate for this reaction was 2 mM *N*-Suc-(Ala)2-Pro-Phe-*p*-nitroanilide (Sigma-Aldrich, STO, St. Louis, MO, USA). The reaction mixture was prepared by mixing 180 µL of the substrate, 120 µL of the supernatant, and 900 µL of 50 mM TRIS-HCl buffer (pH 8.0). The mixture was incubated at 37 °C for 1 h, and then an absorbance reading was performed in a spectrophotometer SM 1100-UV-VIS (Azzota, Claymont, DE, USA) at 405 nm. Enzyme activity is expressed as nanomoles of nitroanilide (NA) released per milliliter per minute at 37 °C.

The substrate used for this reaction was 28 mM pNPB. The reaction mixture consisted of 325 µL of the substrate, 200 µL of the supernatant, and 800 µL of the phosphate buffer (pH 7.25). Next, the mixture was incubated at 37 °C for 5 min, and exactly after that time, a kinetic assay was performed in the spectrophotometer at 405 nm [19,21]. Each enzyme assay was replicated three times using different samples of the four fungal isolates cultivated in different liquid cultures.

### 2.3. Thermostability

The supernatant was incubated at 37 ± 1 °C in a heating chamber for 2, 4, 12, and 24 h. Following the incubation, the supernatant was used for the enzyme assay (as previously described) of the Pr1 and the lipase assay.

### 2.4. Virulence Assay

#### 2.4.1. *Aphis gossypii*

First, all the isolates were cultivated in one-quarter of SDA to produce conidia for the virulence assay, as mentioned previously. Fungal suspensions were prepared at 1 × 10^7^ conidia mL^−1^. The colonies of cotton aphids were provided by the National Institute of Agricultural Sciences, Republic of Korea. They were maintained on three leaf-stage cucumber plants (Ilmi Samcheok, Green Heart Bio, Yeoju, Republic of Korea) grown under laboratory conditions at 26 ± 1 °C, 50 ± 5% relative humidity (RH), and 16:8 h (L:D) photoperiod. All experiments were performed on wingless aphids. Ten aphid nymphs were transferred to a cucumber leaf disc (110 mm diameter). A 1.0 mL aliquot of the fungal conidial suspension was sprayed onto the cucumber leaf discs with the aphids (110 mm diameter) from a distance of 15 cm for 10 sec. The discs were then dried for 10 min at room temperature. Then, the leaves were transferred to disposable plastic cups (50 mL) containing 5 mL of 1% agar (BD Difco, USA). All cups were kept at 26 ± 1 °C, 90 ± 5% relative humidity (RH), and 16L:8D (L:D) cycles. The number of dead aphids was evaluated daily for one week [22]. The mortality was calculated by the proportion of dead aphids compared to the total number of aphids, as demonstrated below:

$$Mortality=\left(\frac{n{}^{\circ}\;of\;dead\;insects}{total\;n{}^{\circ}\;of\;insects}\right)\cdot 100$$

A virulence assay to evaluate the action of the enzymes from the isolate JEF-410 was conducted by forming six groups. These groups included a negative control (Siloxane solution 0.03% (Silwet, FarmHannong Inc., Nonsan, Republic of Korea)), a positive control (Imidacloprid 10% suspension concentrate (SC) (2000×)), fungal conidia, conidia + supernatant, supernatant, and denatured supernatant (boiled for 1 h). The supernatant was produced in an LB broth medium, and aerial conidia were produced on solid one-quarter of SDA medium. The aphids were prepared, and the tests were conducted as mentioned above. The experiment was repeated on three different days.

#### 2.4.2. *Spodoptera frugiperda*

The National Institute of Agricultural Sciences, Republic of Korea, provided the colonies of *S. frugiperda*. The larvae were maintained on a modified artificial diet [23,24], and the insects were maintained at 27 ± 1 °C with a RH of 50 ± 5% and a 14:10 (L:D) photoperiod.

The experiments were prepared as described for the *A. gossypii*, except for the positive control. The *B. bassiana* JEF-492 was tested against the *S. frugiperda* larvae with a negative control (Silwet 0.03%), fungal conidia, conidia + supernatant, supernatant, and denatured supernatant (boiled for 1 h). Five-second instar larvae were transferred to Petri dishes (35 × 10 mm), and a 1.0 mL aliquot of the treatment was sprayed over them from a distance of 15 cm for 10 sec. Next, the larvae were individually transferred to 1% agar cups, and mung bean leaves (*Vigna radiate*) (110 mm diameter) were used as nutritional sources. Mung beans at the three-leaf stage were used in laboratory conditions at 27 ± 1 °C, 50 ± 5% relative humidity (RH), and a 14:10 (L:D) photoperiod. New leaves were provided according to the larvae’s needs. The number of dead larvae was evaluated daily for one week [23]. The mortality was calculated by the proportion of dead larvae compared to the total number of *S. frugiperda* larvae, as demonstrated above with the aphids.

The experiments were repeated on three different days.

### 2.5. Statistical Analysis

All the data were subjected to analysis of variance (ANOVA) and Tukey’s test using BioEstat 5.0 [25]. The *p* values less than 0.05 were considered significant.

## 3. Results

### 3.1. Enzyme Activity

#### 3.1.1. Protease

**Figure 1:**
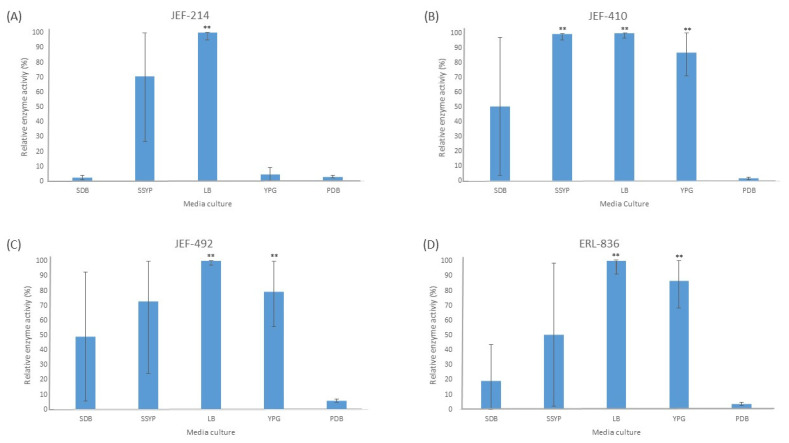
Activity of extracellular Pr1 subtilisin protease. The isolates of *Beauveria bassiana* and *Metarhizium anisopliae* were cultured in SDB (Sabouraud dextrose broth), SSYP (soluble starch–yeast extract–peptone), LB (Luria–Bertani), YPG (yeast extract–peptone–glucose), and PDB (potato dextrose broth) by liquid culture. (**A**) *M. anisopliae* JEF-214; (**B**) *B. bassiana* JEF-410; (**C**) *B. bassiana* JEF-492; and (**D**) *B. bassiana* ERL-836. The cultures were cultivated for 7 days and then filtered and centrifuged to obtain a cell-free supernatant. The vertical bars correspond to the standard error. ** *p* < 0.01 according to the Tukey’s Test.

The liquid culture of all the fungal isolates in different cultured media demonstrated significant activity of the Pr1 protease (Figure 1). The PDB medium did not induce extracellular Pr1 protease significantly among the isolates tested. Furthermore, all the isolates cultured in the liquid LB medium showed a high Pr1 activity, which differed significantly (*p* < 0.01) from the activity in the PDB culture medium. The YPG medium, however, induced Pr1 in the *B. bassiana* isolates (*p* < 0.05) but not in the *M. anisopliae* JEF-214.

#### 3.1.2. Lipase

**Figure 2:**
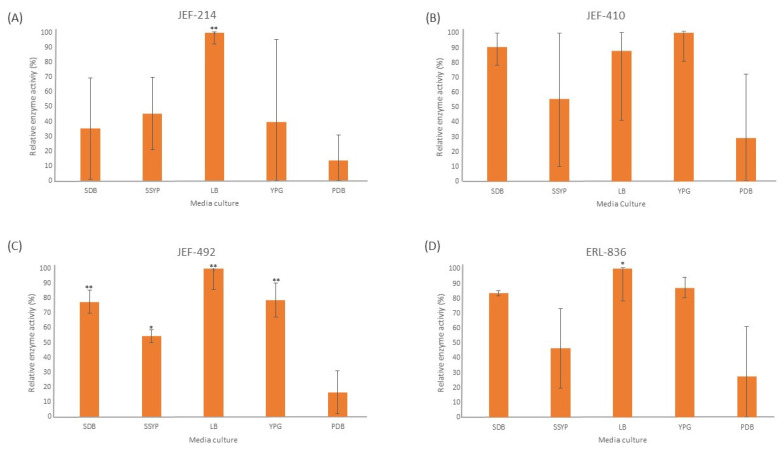
Activity of extracellular lipases. The isolates of *Beauveria bassiana* and *Metarhizium anisopliae* were cultured in SDB (Sabouraud dextrose broth), SSYP (soluble starch–yeast extract–peptone), LB (Luria–Bertani), YPG (yeast extract–peptone–glucose), and PDB (potato dextrose broth) by liquid culture. (**A**) *M. anisopliae* JEF-214; (**B**) *B. bassiana* JEF-410; (**C**) *B. bassiana* JEF-492; and (**D**) *B. bassiana* ERL-836. The cultures were cultivated for 7 days and then filtered and centrifuged to obtain a cell-free supernatant. The vertical bars correspond to the standard error. * *p* < 0.05 and ** *p* < 0.01 according to the Tukey’s Test.

The liquid culture of all the fungal isolates demonstrated significant lipase activity in different cultured media (Figure 2). The LB medium induced a high lipase activity in all the isolates tested, and four out of five isolates differed significantly (*p* < 0.05) from the PDB medium. The isolate JEF-410 was the only isolate with a similar lipase activity in different culture media. The isolate JEF-492 was the only one with a significantly different lipase activity in the SDB, LB, YPG (*p* < 0.01), and SSYP (*p* < 0.05) culture media compared to the activity in the PDB medium.

### 3.2. Thermostability of Supernatant

**Figure 3:**
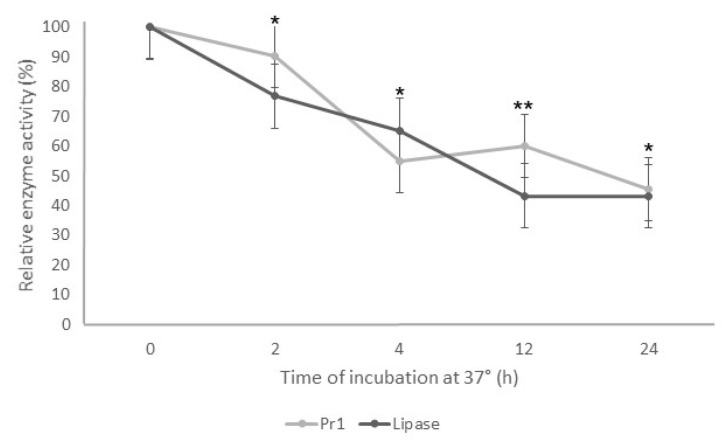
Thermostability of Pr1 and lipases produced by *Beauveria bassiana* JEF-492 after incubation at 37 °C. The isolate was grown in a Luria–Bertani broth medium by liquid culture for 7 days. The supernatant was incubated at 37 °C for 2, 4, 12, or 24 h, and then the protease and lipase activity was evaluated. * *p* < 0.05 and ** *p* < 0.01 according to the Tukey’s Test.

The enzymatic assays decreased the Pr1 and lipase activity when the supernatant was incubated at 37 °C. As the incubation time increased, the enzymatic activity decreased for both enzymes (Figure 3). After 2 h of incubation, both enzymes showed relative activity around 80% and 50% after 4 h. Despite the fast reduction in the protease and lipase activity in the first 4 h incubation, there was a slight difference from 4 to 24 h, with approximately 45% relative activity for both enzymes.

### 3.3. Virulence

#### 3.3.1. *Aphis gossypii*

**Figure 4:**
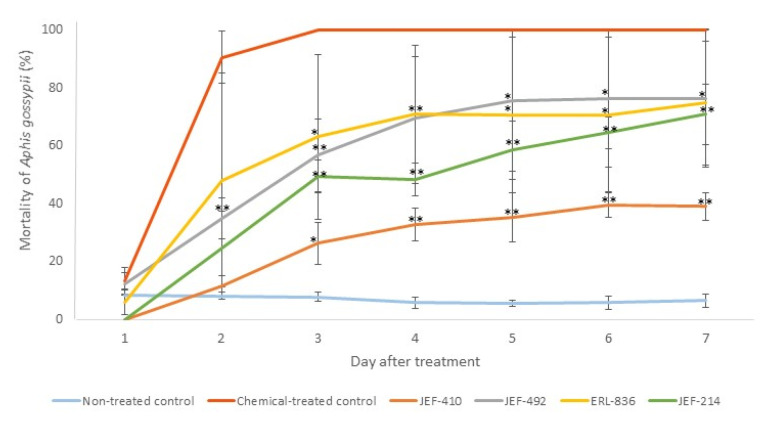
Mortality of *Aphis gossypii* treated with *Beauveria bassiana* and *Metarhizium anisopliae*. The nymphs of the aphids were treated with 1 mL of 1 × 10^7^ conidia mL^−1^ from the *B. bassiana* isolates JEF-410, JEF-492, and ERL-836 and the *M. anisopliae* isolate JEF-214. The isolates were inoculated in one-quarter of SDA for the conidial production. The negative control was 0.03% Silwet (the non-treated control), and the positive control was Imidacloprid (SC) (2000×) (the chemical-treated control). The fungi treatments were only compared to the non-treated control. The analysis was conducted each day after the treatment. * *p* < 0.05 and ** *p* < 0.01 according to the Tukey’s Test.

None of the five isolates caused 100% mortality of the *A. gossypii*. The isolate JEF-410, however, demonstrated the lowest virulence against aphids (Figure 4).

**Figure 5:**
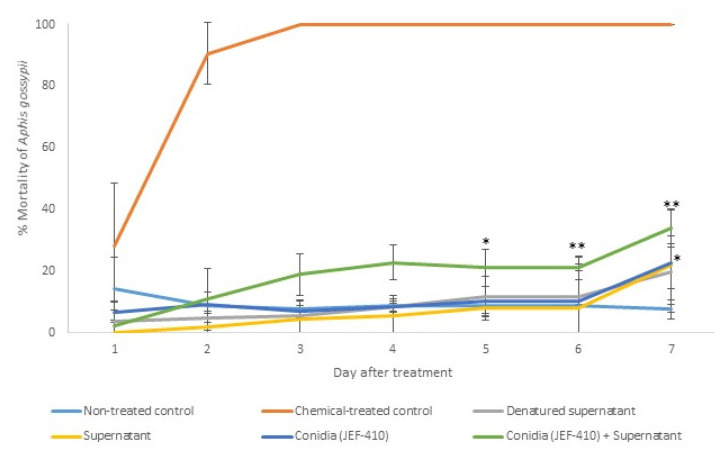
Mortality of *Aphis gossypii* nymphs treated with *Beauveria bassiana* JEF-410. The *B. bassiana* JEF-410 was inoculated in one-quarter of SDA culture medium for the conidial production and the LB broth for the supernatant production. Six treatments were prepared: negative control—0.03% Silwet (non-treated control); positive control—Imidacloprid (SC) (2000×) (chemical-treated control); denatured supernatant (DS); supernatant (S); conidia (C); and conidia + supernatant (CS). One milliliter of the treatment was sprayed over ten nymphs of the aphids, and the number of dead aphids was evaluated daily. The mortality was calculated by the proportion of dead aphids compared to the total number of aphids. The treatments were only compared to the non-treated control. The analysis was conducted each day after the treatment. * *p* < 0.05 and ** *p* < 0.01 according to the Tukey’s Test.

The isolate JEF-410 was selected to investigate the supernatant’s potential to improve fungal virulence and to evaluate whether the supernatant could reduce the mortality rate. The treatment with combined conidia and active supernatant was the only treatment that significantly differed from the negative control 5 and 6 days after the treatment (*p* < 0.05 and *p* < 0.01, respectively). Seven days after the treatment, the groups treated with conidia and conidia + supernatant had a higher mortality than the negative control (*p* < 0.05). The treatments with supernatant combined with fungal conidia caused mortality rates below 50%. The differences between the negative and positive control and the infection caused by JEF-410 are shown in Figure 5.

**Figure 6:**
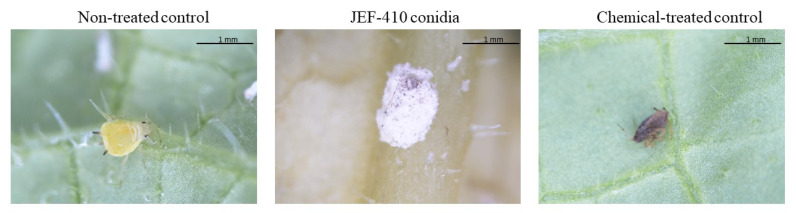
*Aphis gossypii* 7 days after treatment with *Beauveria bassiana* JEF-410. The aphid nymphs were treated with 1 mL of different suspensions: the non-treated control (0.03% Silwet), chemical-treated control (Imidacloprid 10% suspension concentrate (SC) (2000×), and conidia (1 × 10^7^ conidia mL^−1^). The *B. bassiana* JEF-410 was cultured on one-quarter of SDA medium for conidial production.

The difference between the treatment with conidia and the synthetic pesticide is demonstrated in Figure 6.

#### 3.3.2. *Spodoptera frugiperda*

**Figure 7:**
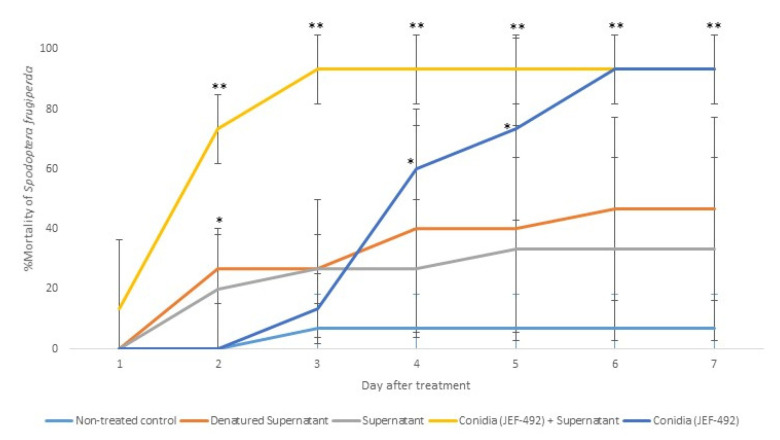
Mortality of *Spodoptera frugiperda* larvae treated with *Beauveria bassiana* JEF-492. The *B. bassiana* JEF-492 was inoculated on one-quarter of SDA medium for the conidial production and the LB broth for the supernatant production. Five treatments were prepared: non-treated control (0.03% Silwet); denatured supernatant (DS); supernatant (S); conidia (C); conidia + supernatant (CS). One milliliter of the treatment was sprayed over five-second instar larvae, and the number of dead larvae was evaluated daily. The mortality was calculated by the proportion of dead larvae compared to the total number of larvae. The conidia and supernatant treatments were only compared to the non-treated control. The analysis was conducted each day after the treatment. * *p* < 0.05 and ** *p* < 0.01 according to the Tukey’s Test.

Previous laboratory studies demonstrated that the isolate JEF-492 is virulent against *S. frugiperda* [23]. Therefore, we evaluated the supernatant’s potential to increase the fungal virulence. The treatment with conidia + supernatant resulted in higher and faster mortality than using only conidia (Figure 7).

**Figure 8:**
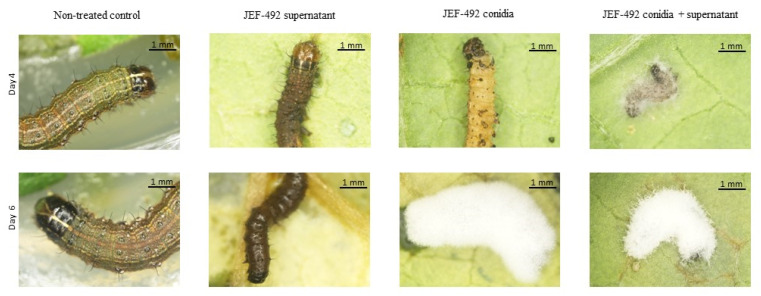
*Spodoptera frugiperda* larvae after treatment with *Beauveria bassiana* JEF-492. Five second instar larvae were treated by spraying with 1 mL of different suspensions: the non-treated control (0.03% Silwet); supernatant; conidia (1 × 10^7^ conidia mL^−1^), or the conidia suspended in supernatant (1 × 10^7^ conidia mL^−1^). Four days after the treatment, the larvae treated with the supernatant showed a dark brown coloration. The conidia treatment showed dead larvae but no mycelium growing over the insect cadaver. The treatment with conidia + supernatant showed dead larvae and mycelium growing over the insect cadaver. The insect treated with conidia or conidia + supernatant showed growing fungal mycelium on day 6 after treatment.

The group treated with conidia and supernatant showed fungal sporulation in a shorter period than those treated only with conidia (Figure 8). Although the denatured supernatant and supernatant had significant mortality only on day 2 after the treatment, the dead larvae showed a dark brown color. This suggests a possible secondary metabolite toxicity causing larval mortality in both treatments. The treatments with conidia + supernatant caused significant mortality starting on day 2 and sporulation on day 4. By day 6 after treatment, the fungal mycelium covered the cadaver completely.

## 4. Discussion

The culture media impact fungal growth differently. The Luria–Bertani broth medium induced a high quantity of extracellular enzymes, such as Pr1 protease and lipases, for *Beauveria bassiana* and *Metarhizium anisopliae* isolates [19]. Different fungal isolates produce different quantities of enzymes in the same liquid culture media, so it is crucial to examine the impact of the culture media on each fungal isolate to optimize enzyme production.

When the fungus is cultivated in liquid culture media containing simple compounds, such as carbohydrates, it does not induce the production of enzymes involved in the infection process. In contrast, a medium that does not offer simple carbohydrates induces the production of extracellular enzymes [26]. Starvation conditions and complex compounds in the culture medium enhance the virulence of entomopathogenic fungi and induce the production of enzymes, such as chitinases, proteases, and lipases [26,27].

Fungal isolates may exhibit a distinct virulence towards specific insect species. The insect cuticle serves as the primary barrier against the penetration of external compounds. It consists of different layers of lipids, proteins, hydrocarbons, and chitin fibers. The cuticle composition, however, varies according to age, sex, insect order, and environmental conditions [28]. The virulence of the fungal isolates depends on the ability to produce different isoforms capable of degrading the host cuticle, especially the protease Pr1, which contains 11 isoforms [10,29]. In this study, a screening was performed to evaluate the virulence of different fungal isolates against *A. gossypii* nymphs. The least virulent isolate, JEF-410, was selected to assess whether the treatment with conidia associated with their supernatant-containing enzymes would increase insect mortality; however, the highest mortality did not reach 50% after 7 days. Regardless of the number of enzymes applied in combined treatments with conidia, no significant increased mortality of *A. gossypii* nymphs was detected.

The opposite strategy was applied to treat *S. frugiperda*. *B. bassiana* JEF-492, a previously known virulent isolate, was tested in this study. In this case, when the conidia were associated with the supernatant, there was a significant reduction in the host infection time compared to the conidia treatment. This result reinforces the effectiveness of the enzyme isoforms in degrading the hosts’ cuticle. An increase in the quantity of these isoforms facilitated the infection process of the *B. bassiana* JEF-492 against the *S. frugiperda* larva.

The supernatant (i.e., crude extract or crude enzymes) from the fungal fermentation has the potential to be used as biopesticides [7]. The supernatant contains several molecules, including enzymes, other proteins, and secondary metabolites. All these components can contribute to the toxicity of the supernatant against insects. The insecticidal activity against *S. litura* larvae of the supernatant from the *B. bassiana* fermentation has already been demonstrated. The toxicity of the supernatant from the fungal fermentation was possibly due to the enzymes and metabolites [30]. This proposition is supported by studies that showed that the protein extract from the supernatant of entomopathogenic fungi causes lysis in the midgut epithelium and deterioration of the microvilli in *S. littoralis* larvae [31]. All these data are consistent with the results obtained in this current study, which demonstrated a decreased time of infection of the *S. frugiperda* larvae treated with the *B. bassiana* conidia combined with the supernatant from the fungal fermentation than the larvae treated with conidia only. This reinforces that the supernatant from the fungal fermentation can be used for the biological control of pests without requiring enzyme purification.

One of the biggest challenges of applying fungal enzymes for pest control is their low thermostability. This present study demonstrated that Pr1 and lipases still have high activity—around 80%—after 2 h at 37 °C. This is compatible with the results obtained from a previous study [32], which demonstrated that fungal alkaline protease had more than 80% residual activity after exposure at 40 °C for a 1 h incubation. Nonetheless, the residual activity of alkaline proteases decreased to 40% at 50 °C. Entomopathogenic fungi, in general, have an optimum radial growth between 25 °C and 30 °C—with a maximum thermal threshold at 37 °C for *B. bassiana* [33,34]. Considering that conidia are successfully applied in the field, the temperature in the application area is below 40 °C, indicating that the enzymes would not be denatured immediately. These findings suggest that the enzymes Pr1 and lipases exhibit substantial activity at 37 °C after 2 h and maintain significant activity after 24 h. This may be sufficient time to cause damage to the cuticle of immature insects, as observed for the second instar larvae of *S. frugiperda*. Additionally, a proper formulation can stabilize the enzyme activity at high temperatures for extended periods. This can be achieved, for example, using calcium chloride and glycine [35] or deep eutectic solvents [36].

## 5. Conclusions

The results of this study demonstrated that the extracellular enzymes produced by entomopathogenic fungi can be combined with conidia to reduce the time of infection, as demonstrated for *S. frugiperda* larvae. Moreover, our results indicate that the supernatant from the fermentation of entomopathogenic fungi has the potential to be used in new biopesticide formulations, providing efficient strategies for the biological control of insect pests.

## 6. Patents

All the isolates evaluated in this work are patented in Republic of Korea. The patent registration numbers are JEF-410: KR 10-2105252; ERL-836: KR 10-1626801; and JEF-214: KR 10-2054577; and the patent application number is JEF-492: KR 10-2022-0074643.

## Data Availability

Data are contained within the article.
